# Hyperthermia suppresses the biological characteristics and migration of chicken primordial germ cells

**DOI:** 10.3389/fgeed.2024.1512108

**Published:** 2025-01-08

**Authors:** Yuzhou Gu, Kexin Wu, Bowen Niu, Zhiting Wang, Yuchen Jie, Zixuan Fan, Junying Li, Congjiao Sun, Zhuo-Cheng Hou, Li-Wa Shao

**Affiliations:** ^1^ State Key Laboratory of Animal Biotech Breeding, China Agricultural University, Beijing, China; ^2^ National Engineering Laboratory for Animal Breeding, College of Animal Science and Technology, China Agricultural University, Beijing, China; ^3^ Frontiers Science Center for Molecular Design Breeding (MOE), China Agricultural University, Beijing, China

**Keywords:** gene-edited chicken, primordial germ cell, hyperthermia, cell biological characteristics, migration

## Abstract

Primordial germ cells (PGCs) play a crucial role in transmitting genetic information to the next-generation. In chickens, genetically edited PGCs can be propagated *in vitro* and subsequently transplanted into recipient embryos to produce offspring with desired genetic traits. However, during early embryogenesis, the effects of external conditions on PGC migration through the vascular system to the gonads have yet to be explored, which may affect the efficiency of preparing gene-edited chickens. In this study, we investigated the effects of hyperthermia on the biological characteristics and migration of chicken PGCs. A gonad-derived PGC line of White Leghorn (WLH) chicken was established and verified through PAS staining and immunofluorescence of PGC-specific proteins. To visually observe PGC migration *in vivo*, GFP-positive PGCs were prepared and locations of chimeras were validated. Cell viability, glycogen granule contents, and mRNA expression levels of pluripotency markers (*NANOG* and *POUV*), germ cell-specific markers (*DAZL* and *CVH*), and telomerase reverse transcriptase (*TERT*) were reduced in PGCs cultured under high temperatures (43°C for 12, 24, and 48 h). After the heat treatment of donor PGCs (43°C) or recipient embryos (39.5°C), GFP-positive PGCs in gonads were rarely observed. Taken together, our results underscore the negative effects of hyperthermia on the biological characteristics and migration of chicken PGCs, which provides valuable insights for the implementation of PGC-based gene editing techniques in chickens.

## 1 Introduction

Genetic editing in chickens is crucial for advancing poultry science and agriculture. It allows for the precise modification of the chicken genome to explore specific gene functions or introduce desirable traits, such as disease resistance and improved growth efficiency. However, the process of preparing genetically edited chickens is fraught with challenges due to the unique reproductive physiology of chickens and the structural complexities of their ova and early embryos. Fertilized chicken eggs undergo rapid cell division in the oviduct, resulting in a large number of cells by the time the egg is laid, which complicates the procurement of early embryos for genetic editing operations. The substantial yolk content and the shell of chicken eggs pose physical barriers to microinjection, and these factors also render the cryopreservation of chicken oocytes, zygotes, or early embryos technically challenging. Traditional methods to deliver genome editors into chicken embryos, such as direct electroporation, blastoderm injection, and embryonic vascular system injection, have been employed but are characterized by low efficiency (Rieblinger et al., 2021; Agate et al., 2009; Tyack et al., 2013; Challagulla et al., 2023). Chicken sperm can be directly transfected and used for artificial insemination, but the number of offspring resulting from genetic modification is quite low (Cooper et al., 2017).

Utilizing primordial germ cells (PGCs) presents a comparatively effective approach for genome editing in chickens. PGCs are the precursors of male and female germ cells, and the genetic information they carry is passed on to offspring through sexual reproduction (van de Lavoir et al., 2006). Chickens are among the few species in which PGCs can be isolated from early embryos and propagated *in vitro* using a specific culture medium (Jung et al., 2017; Whyte et al., 2015; Dehdilani et al., 2023; Naito et al., 2015). By employing viruses or transposition systems, genome editing technologies such as the clustered regularly interspaced short palindromic repeat (CRISPR) and CRISPR-associated protein 9 (Cas9) systems allow for precise editing of genetic information within chicken PGCs. Upon microinjection of exogenous gene-edited PGCs into chicken embryos, these cells can successfully colonize the gonads of the recipient chickens, thereby generating offspring with desired traits. (Jin et al., 2022; Idoko-Akoh and McGrew, 2023; Han and Lee, 2023). This PGC-based method enables the direct introduction of genetic modifications into the germline and provides a more controlled environment for genetic manipulation. As a result, it leads to higher success rates and more predictable outcomes in the field of avian gene editing. For instance, Lee et al. developed chicken sex determination models by utilizing PGCs with disrupted DMRT1 genes (Lee et al., 2021). Ballantyne et al. created inducibly sterile chickens by utilizing iCaspase9 integrated PGCs (Ballantyne et al., 2021). Idoko-Akoh et al. successfully generated influenza-resistant chickens by using ANP32A-edited PGCs (Idoko-Akoh et al., 2023). Kinoshita et al. successfully generated eumelanin reduced chickens by using TYRP1-edited PGCs (Kinoshita et al., 2024). Currently, the technical challenges in preparing gene-edited chickens based on PGCs include the instability of PGC gene editing efficiency, particularly the extremely low efficiency of HDR (homology-directed repair); the low chimerism ratio of donor PGCs in the gonads of recipient chicken embryos; the uncertainty of the ability of donor PGCs in the gonads to successfully differentiate into functional gametes; and the impact on the hatching rate of fertilized eggs after injection procedures.

The biological characteristics and precise migration capabilities of donor PGC cells determine their chimerism rate in the gonads and their ability to differentiate into germ cells, which in turn directly affects the success rate of preparing gene-edited offspring. In fertilized eggs during hatching, chicken PGCs are originally found in the center of the zona pellucida in the blastoderm, accumulate in the germinal crescent at Hamburger–Hamilton (HH) stage 4, migrate to gonads through the blood circulation beginning at HH12, and finally colonize embryonic gonads at about HH28 (Ichikawa and Horiuchi, 2023). Thus, PGCs are typically isolated from embryonic blood after 2.5 days of incubation or from embryonic gonads after 5.5 days of incubation (Yu et al., 2019; Altgilbers et al., 2021). Utilizing the PGC-based germline transmission method for generating genetically modified chickens, exogenous PGCs are transplanted into embryos after 2.5 days of incubation. These PGCs then undergo approximately 4 days of migration before colonizing the embryonic gonads. The migration of donor PGCs within avian embryos is a complex process regulated by multiple factors. As egg-laying species, the hatching process from embryo to chicks outside of the mother’s body could be affected by many external factors, such as temperature, humidity, and egg turning (Noiva et al., 2014; Oliveira et al., 2020). Incubation temperature, a critical physical factor, may affect the hatching rate and chick quality by changing the utilization of egg nutrients and rate of embryonic development (Dayan et al., 2020; Joseph et al., 2006). Chicken eggs normally hatch at temperatures between 37°C and 38°C, but conditions such as power failures or summer heatwaves can lead to uncontrollable temperature increases within the incubator. While the effects of hyperthermia during chicken embryogenesis on developmental stability, hatchability, and post-hatching performance have been investigated extensively (Narinç et al., 2016; Maatjens et al., 2016; Oksbjerg et al., 2019; Yalcin et al., 2022), the specific impacts of hyperthermia on chicken PGC characteristics and PGC migration within the embryo remain understudied.

Here, we established a chicken PGC line through embryonic gonad isolation, cell propagation, and PGC identification. Using this cell line, we investigated the effects of heat treatment on the biological characteristics of chicken PGCs *in vitro*, including glycogen granules, pluripotency, and reproductive chimerism. We generated GFP-positive PGCs through cell transfection and screening, and used them to visually assess whether hyperthermal treatment affected PGC migration in chicken embryos. This study elucidates the impact of hyperthermia on chicken PGCs and their migration within embryos, offering valuable data for future studies and applications of PGC-based gene editing in chickens.

## 2 Materials and methods

### 2.1 PGC isolation and culture

For gonad-derived PGC isolation and culture, previously described methods were used, with some modifications (Whyte et al., 2015; Altgilbers et al., 2021). 80 fresh fertilized White Leghorn eggs (Beijing Boehringer Ingelheim, Beijing, China) were incubated at 37.8°C and 65% relative humidity for 5.5 days. The gonads dissected from embryos were digested by 0.25% Trypsin/EDTA at 37°C for about 10 min and gently triturated with a pipette approximately 40 times. After centrifugation at 500 × *g* for 5 min, the cells were transferred to a 24-well plate containing PGC medium (Avian KO-DMEM basal medium, 100 µM β-mercaptoethanol, 100 µM Sodium Heparin, 10 µg/mL Ovotransferrin, 0.2% Ovalbumin, 4 ng/mL rhFGF, 30 ng/mL Activin A, 1 × B-27 supplement) for 3 h. Then, the supernatant PGCs were transferred to a 48-well plate. To establish the PGC cell line, gonad-derived cells were culture in PGC medium for at least 3 weeks and the medium was refreshed every other day.

### 2.2 PAS staining

PAS staining of PGCs was performed using the PAS Staining Kit (G1360; Solarbio, Beijing, China) according to the manufacturer’s instructions. PGCs cultured on poly-lysine-coated slides (WHB-24-CS-LC; WHB, Shanghai, China) were fixed using fixative for 10–15 min, oxidized using an oxidant for 15–20 min, and then washed in water four times. When dry, the slides were dipped in Schiff reagent for 10–20 min, washed slowly with sodium sulfite solution twice for 2 min each time, and rinsed with running water for 5 min. After staining in Mayer’s hematoxylin solution for 1–2 min, the PGCs on slides were subjected to microscopic observation and imaging.

### 2.3 Immunofluorescence

PGCs cultured on poly-lysine-coated slides were fixed in 4% paraformaldehyde at room temperature for 10–30 min. After they were washed once with PBS, cells were permeabilized with 0.2% Triton-X100 on ice for 20 min and blocked with 10% FBS at room temperature for 0.5–1 h. Then, the PGCs were incubated at 4°C overnight with designated primary antibodies (anti SSEA-1 antibody: ab16285, 1:500, Abcam, Cambridge, United Kingdom; anti DAZL antibody: 12633-1-AP, 1:100, Proteintech, Rosemont, IL, United States) and incubated at room temperature for 1 h with designated secondary antibodies. After staining with DAPI (A0568, 1:500; Beyotime, Shanghai, China) for 5 min, images of PGCs were obtained using a confocal microscope (A1 HD25; Nikon, Tokyo, Japan).

### 2.4 Generation of GFP-positive PGCs

PB-EGFP and PBase plasmids were a generous gift from Dr Sen Wu. Plasmids were transfected into cells using Fugene HD Reagent (E2311; Promega, Madison, WI, United States) and Opti-MEM (31985062; Gibco, Carlsbad, CA, United States). GFP expression in PGCs was observed after 48 h. Then, the PGCs were treated with 0.5 µg/mL puromycin to screen out all GFP-positive cells in 24–48 h.

### 2.5 Visualization of PGC migration and colonization

The experiment was performed following previously described methods (Jin et al., 2022). Fresh fertilized WLH eggs were incubated for 2.5 days at 37.8°C and 65% relative humidity. Then, eggshell surfaces were sterilized and each blunt end was tapped out to form a window to expose the embryo. Male GFP-positive PGCs filling a glass needle were injected into dorsal aorta of either male or female embryo under a microscope. For each embryo, approximately 3,000 cells were transplanted. Next, 200 µL of Penicillin/Streptomycin solution was added into the egg. The broken window was sealed with cling wrap, and the egg was placed back in the incubator. After 6 or 9 days of development, the recipient embryos or gonads were isolated, and the locations of GFP-positive PGCs were evaluated using a stereoscopic fluoroscope (Leica, Leica Thunder, Wetzlar, Germany).

### 2.6 Heat treatment of PGCs and chicken embryos

The temperatures used to simulate heat stress in chicken cell cultures range from 42°C to 45°C. Based on previous studies (Ibtisham et al., 2018; Sun et al., 2015; Slawinska et al., 2016; Xu et al., 2017), PGCs were subjected to different temperatures and various durations, and the final hyperthermia condition for cells was determined based on the upregulation of heat shock proteins (HSPs) and the viability that remained relatively unaffected. Similarly, based on previous research (Narinç et al., 2016; Dayan et al., 2020), WLH eggs were subjected to different temperature treatments at various stages during the incubation period. The hyperthermia condition for recipient embryos was ultimately determined based on the hatchability and chick development phenotypes that were not severely affected.

To prepare heat-treated (HT) PGC samples, PGCs in 6-well plates were treated at 43°C in a cell incubator for 0, 12, 24, and 48 h. To prepare HT samples of chicken embryos, fertilized eggs were hatched at 39.5°C in an egg incubator since embryonic day (ED) 4 to embryonic day 9. Except for these HT periods, eggs hatched normally at 37.8°C.

All animal experiments in this study were approved by the Animal Care and Use Committee of China Agricultural University.

### 2.7 CCK-8 assay

PGC proliferation was assessed using the CCK-8 Kit (C0037: Beyotime, Shanghai, China), following the manufacturer’s protocol. Preliminary experiments were conducted to establish optimal incubation periods and cell densities for the subsequent formal experiments. Sufficient PGCs were prepared, and cells were resuspended in 100 µL of medium per well at varying densities (2000, 4,000, 8,000, 16,000 cells/well). Then, 10 µL of CCK-8 reagent was added to each well and incubated for different durations (1, 1.5, 2, 2.5, and 3 h). The CCK-8 reaction yields a bright orange color indicative of proliferating cells, with absorbance measurable at 450 nm. Data were plotted and subjected to linear regression analysis to identify the optimal cell density and incubation time for the formal experiments. The pre-experiments determined that an optimal cell density of 8,000 cells per well and an incubation time of 1.5 h were most suitable. To assess cell viability under defined conditions, an equal number of PGCs from each sample were seeded into a 96-well plate (6 wells per sample) and incubated with 10 µL of CCK-8 solution at 37°C for 1.5 h. Cell viability was determined by correlating the number of proliferating cells with the absorbance at 450 nm.

### 2.8 RNA isolation and real-time PCR

PGCs were collected and re-suspended in TRIzol reagent (15596018CN; Invitrogen, Carlsbad, CA, United States). Total RNA was isolated by chloroform extraction, precipitated with isopropanol, and washed with 75% (v/v) ethanol (Toni et al., 2018). cDNA was synthesized using the PrimeScript™ RT Reagent Kit (RR047A; Takara, Tokyo, Japan). Quantitative PCR was performed using TB Green^®^ Premix Ex Taq™ (RR420A; Takara). For quantification, PGC transcripts were normalized to *GAPDH* levels. Primer sequences for quantitative RT-PCR are provided in Supplementary Table 1.

### 2.9 Statistical analysis

Statistical analyses were performed using GraphPad Prism 9.0 (GraphPad Software, La Jolla, CA, United States). Results are expressed as the mean ± SEM. One-way ANOVA was used for comparisons among groups. All experiments were performed independently at least twice with similar results, and representative images are shown in the figures.

## 3 Results

### 3.1 Biological characteristics of the chicken primordial germ cell line

**FIGURE 1:**
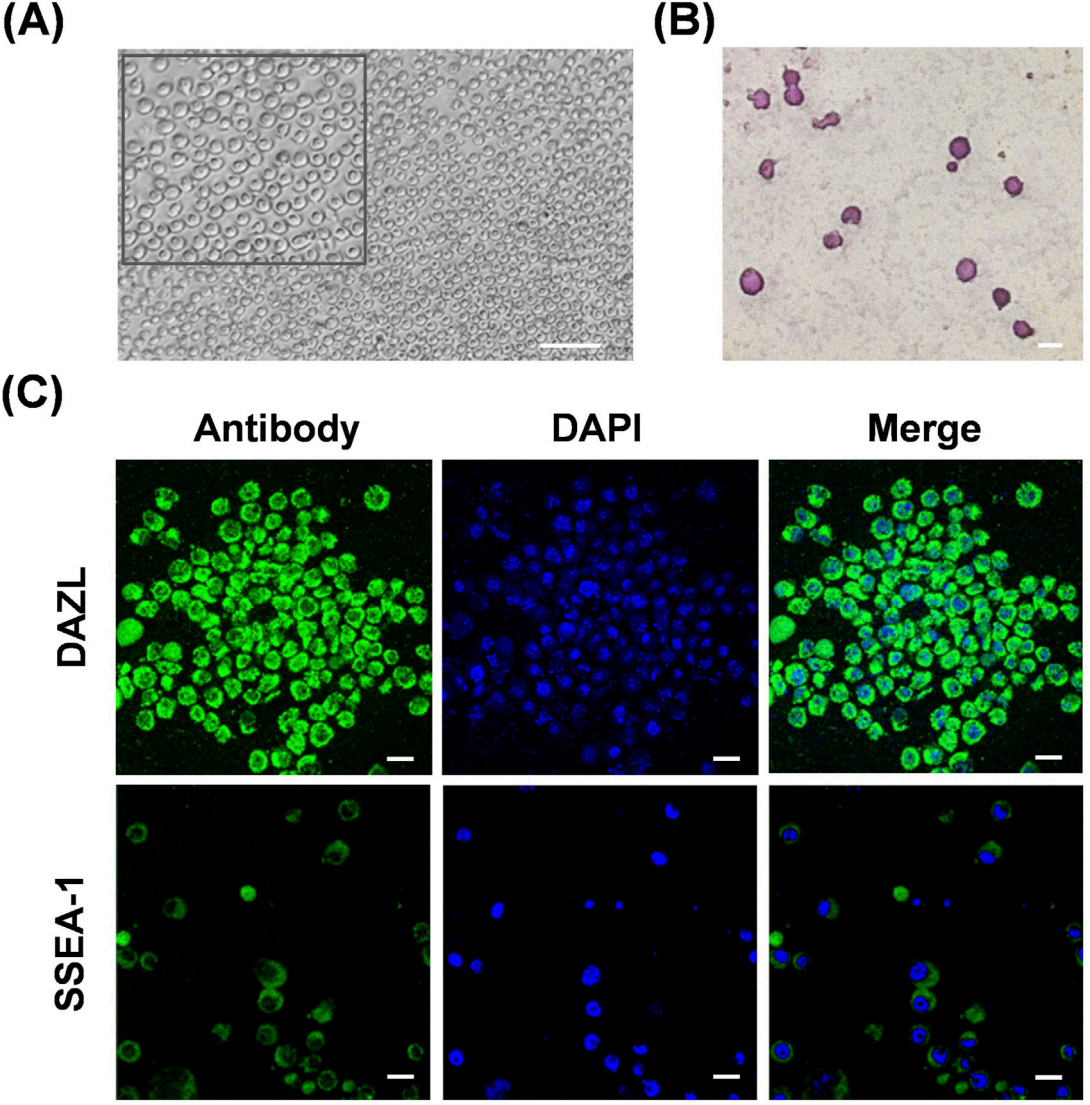
Characterization of the chicken primordial germ cell line. **(A)** Representative images of cultured chicken PGCs *in vitro*. An enlarged image is shown in the top box. Scale bar, 100 μm. **(B)** PAS staining of cells from the chicken PGC line. Scale bar, 20 μm. **(C)** Immunofluorescence staining of DAZL or SSEA-1 in cells from the chicken PGC line. Scale bar, 20 μm.

To reveal the effects of hyperthermia on chicken PGC migration *in vivo*, we initially isolated PGCs from embryonic gonads of WLH to generate a cell line. Cells cultured *in vitro* were suspended as single transparent spheres (Figure 1A). Chicken PGCs reportedly contain high levels of glycogen granules, which can be dyed red using periodic acid-Schiff (PAS) staining, and they are known for their pluripotency (Macdonald et al., 2010). PAS staining verified the establishment of the PGC line (Figure 1B). Using immunofluorescence, we confirmed the expression of the germ cell-specific marker DAZL in the cytoplasm of the PGC line, and the presence of the stem cell-specific marker SSEA-1 on the cell surfaces of the PGC line (Figure 1C). Collectively, these data validate the biological characteristics of the gonad-derived WLH PGC line.

### 3.2 Visualization of chicken PGC migration and colonization in embryos

**FIGURE 2:**
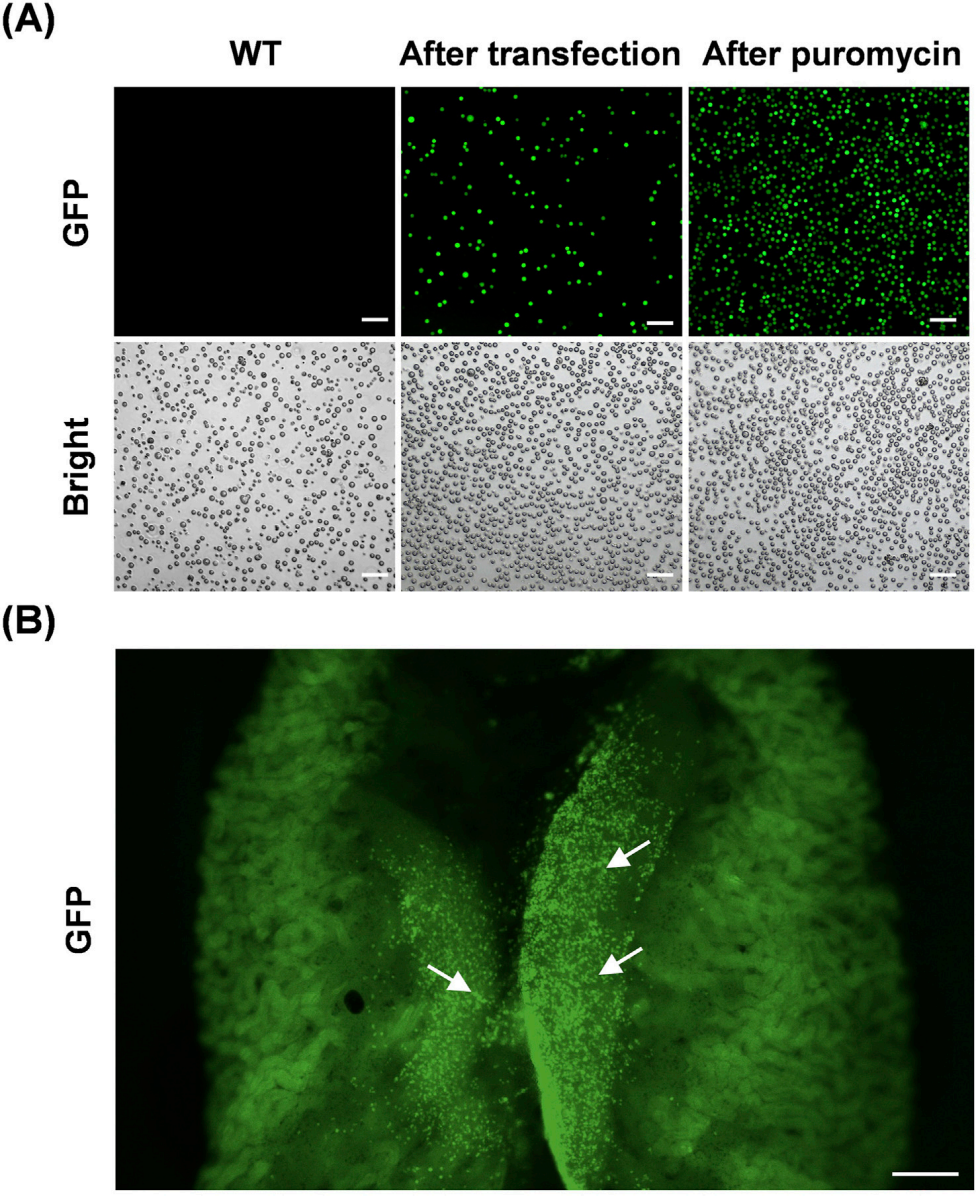
Visualization of PGC migration and colonization *in vivo*. **(A)** Representative images of chicken PGCs during the process of establishing a GFP-expressing cell line *in vitro*. Scale bar, 100 μm. **(B)** Colonization of recipient chicken embryonic gonads by donor GFP-positive PGCs. The arrows in the image indicate representative GFP-positive PGCs. Scale bar, 500 μm.

To verify the ability of cultured PGCs to generate germline chimeras and to visually detect PGC migration and colonization during early embryogenesis, we transplanted male GFP-positive PGCs into the dorsal aortas of recipient chicken embryos. PGCs with green fluorescent protein expression were obtained by transfection in *GFP* plasmids; however, the proportion of positive cells was <30% (Figure 2A). Subsequently, puromycin screening was used to obtain cells expressing *GFP* (Figure 2A). Approximately 3,000 GFP-positive PGCs were microinjected into each embryo. As chicken PGCs migrate through blood flow beginning at HH12, 2.5 days recipient embryos were injected. Then, after 6 days of incubation, gonads containing donor GFP-positive PGCs were observed in the dissected male or female embryos (Figure 2B). These results demonstrate the chimeric ability of cultured WLH PGCs and indicate that this visual system can be applied to explore the migration and settlement of PGCs *in vivo*.

### 3.3 Effects of hyperthermia on chicken PGCs *in vitro*

**FIGURE 3:**
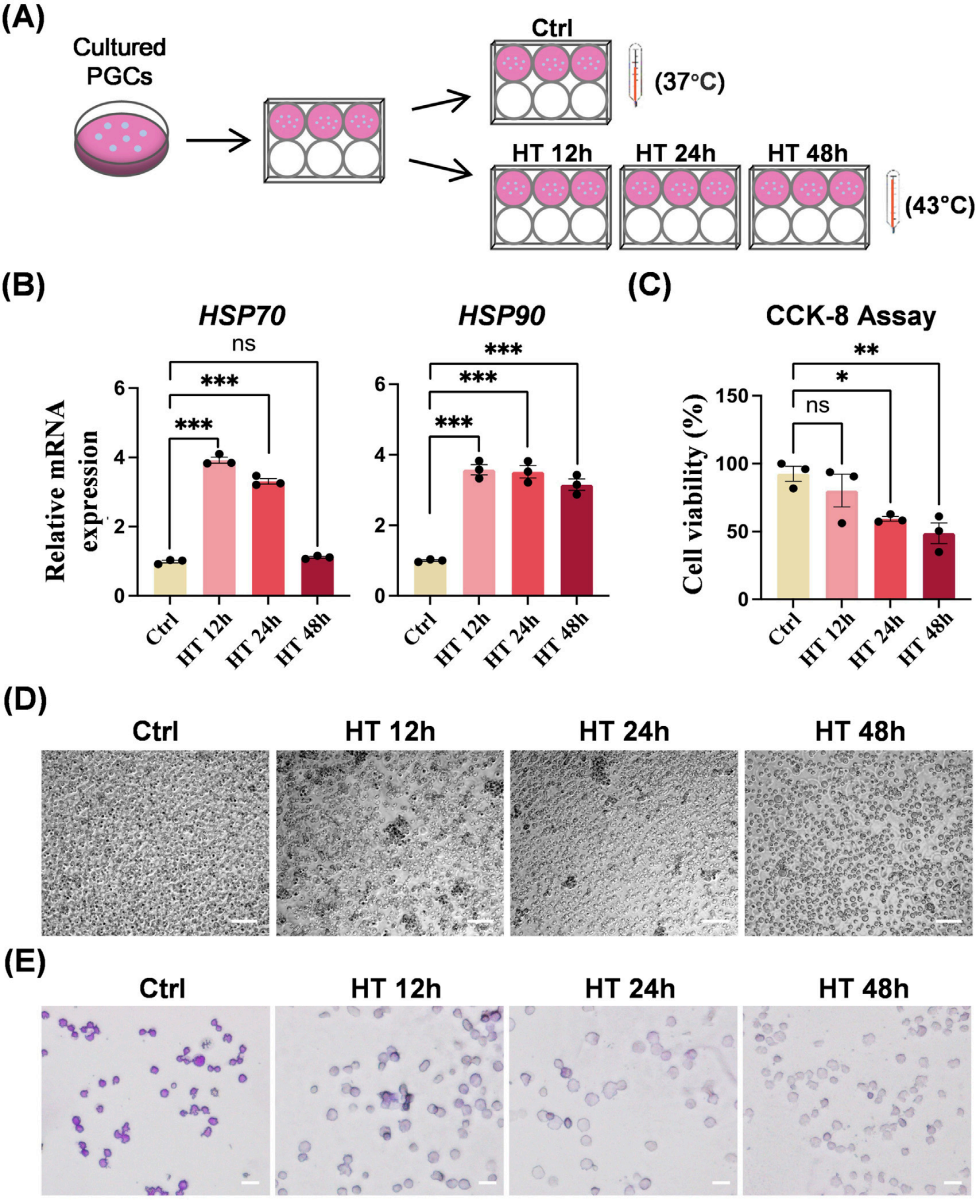
Hyperthermia affects biological characteristics of chicken PGCs *in vitro*. **(A)** Schematic of control (ctrl) PGCs cultured at 37°C or heat-treated PGCs cultured at 43°C for 12 h (HT 12 h), 24 h (HT 24 h), or 48 h (HT 48 h). **(B)** qRT-PCR analyses of mRNA levels of *HSP70* and *HSP90* in PGCs cultured under the indicated conditions. ***P < 0.001, ns (not significant) *P* > 0.05. **(C)** CCK8 assay of the viability of PGCs cultured under the indicated conditions. *P < 0.05, **P < 0.01. **(D)** Representative images of PGCs cultured under the indicated conditions. Scale bar, 100 μm. **(E)** PAS staining of PGCs cultured under the indicated conditions. Scale bar, 20 μm.

We subcultured equal numbers of PGCs from the same plate of the WHL PGC line into 6-well plates and placed each plate in incubator. Cells in the control (Ctrl) group were cultured at 37°C throughout the whole process, and cells in HT groups were cultured at 43°C for 12, 24, and 48 h (Figure 3A). The heat shock response, a vital cellular defense mechanism, involves the activation of *HSPs* such as *HSP70* and *HSP90* in response to diverse stressors. Heat treatment in HT groups triggered this response, leading to an almost complete upregulation of the mRNA expression of *HSP70* and *HSP90* (Figure 3B). Besides, heat treatment suppressed cell viability significantly (Figures 3C, D). The longer the duration of heat treatment, the lower the survival of PGCs. The dead PGCs in HT groups cause cells aggregation. Moreover, PAS staining showed fewer glycogen granules in PGCs in HT groups than in the Ctrl group (Figure 3E).

**FIGURE 4:**
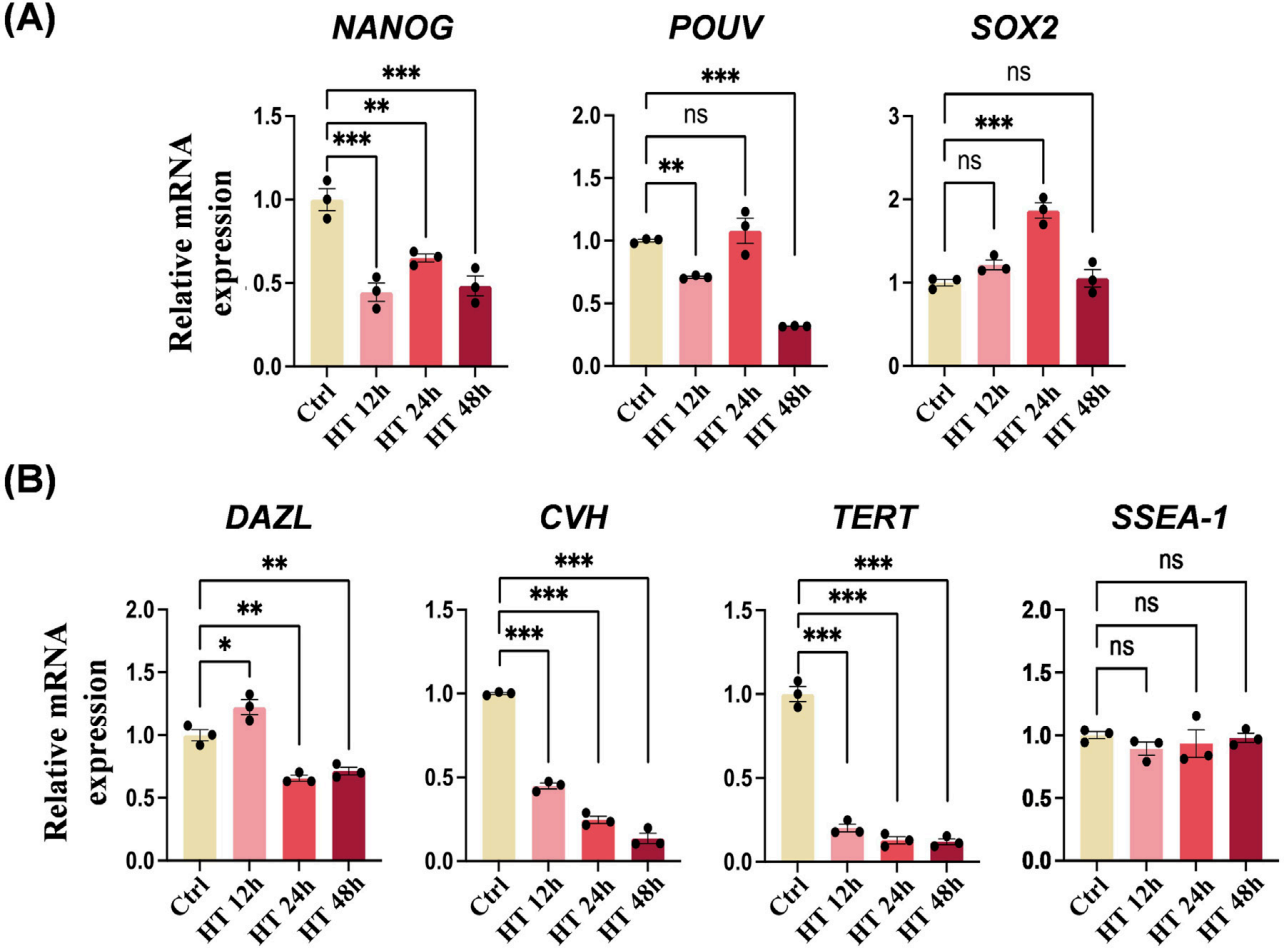
Hyperthermia affects the expression of PGC-specific genes. **(A)** qRT-PCR analyses of mRNA levels of *NANOG*, *POUV*, and *SOX2* in PGCs cultured under the indicated conditions. **P < 0.01, ***P < 0.001. **(B)** qRT-PCR analyses of mRNA levels of *DAZL*, *CVH*, *TERT*, and *SSEA-1* in PGCs cultured under the indicated conditions. *P < 0.05, **P < 0.01, ***P < 0.001.

We further examined the expression profile of PGC-specific genes in Ctrl and HT cells. The mRNA expression levels of pluripotency marker genes varied (Figure 4A). *NANOG* was suppressed in all HT groups with different heat durations, *POUV* was suppressed in the HT 12 h and HT 48 h groups, and *SOX2* was not suppressed and was even activated in the HT 24 h group. In addition, the mRNA expression levels of germ cell-specific marker genes (*DAZL* and *CVH*) and telomerase reverse transcriptase (*TERT*) were all suppressed by hyperthermia, whereas *SSEA-1* was not affected (Figure 4B). Taken together, these results indicate that hyperthermia in cultured chicken PGCs induces a heat shock response, lowers viability, suppresses germ cell specificity, and partially alters pluripotency.

### 3.4 Effects of hyperthermia on chicken PGC migration *in vivo*

**FIGURE 5:**
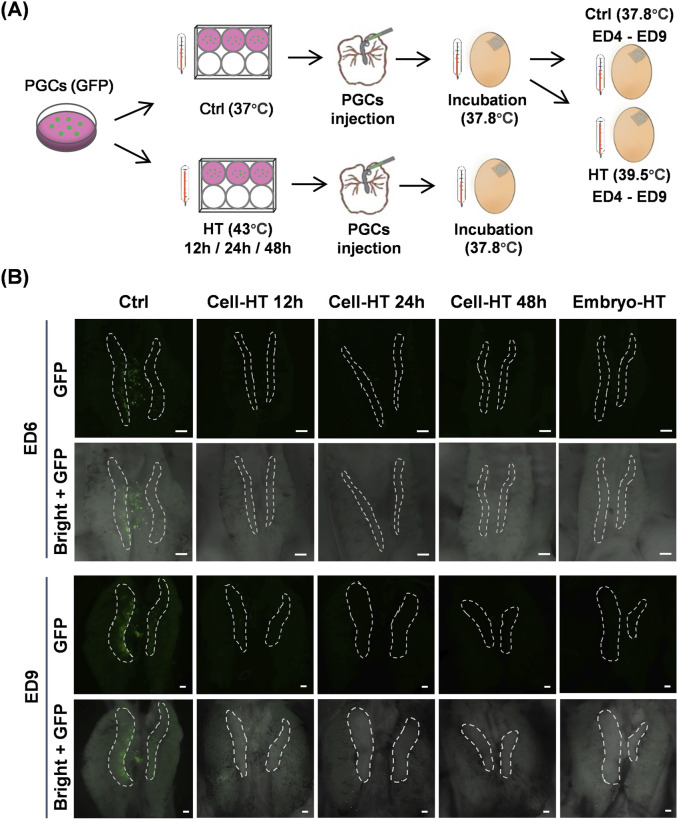
PGC migration *in vivo* was inhibited by hyperthermia. **(A)** Schematic of the preparation of chimeric chicken embryos by micro-injecting donor GFP-positive PGCs into recipient embryos with PGCs that were heat-treated before injection or embryonic heat treatment after injection. **(B)** Representative images showing donor GFP-positive PGCs in recipient embryos under the indicated conditions. White dashed circles indicate gonads. Scale bar, 200 μm. (N = 3 independent experiments, n = 8 embryos per group).

To uncover the influence of hyperthermia on PGC migration in embryos, we observed the location of donor GFP-positive PGCs inside the early embryo using heat-treated donor PGCs or recipient embryos (Figure 5A). PGCs were transplanted into embryos on ED2.5, and stereo fluorescence detection was performed on ED6 and ED9. In HT groups, PGCs were cultured at 43°C for 12, 24, or 48 h, and embryos were incubated at 39.5°C during ED4–ED9. Notably, we found that on ED6, the donor PGCs in the control group remained in close proximity to the embryonic gonad, whereas the donor PGCs in HT groups were nearly undetectable (Figure 5B). On ED9, the donor PGCs in the control group predominantly localized in the embryonic gonad, whereas in the HT groups, the donor PGCs were still rare and only a few cells in the HT 12 h group were detected near the embryonic gonad (Figure 5B). Collectively, these results suggest that either hyperthermia in PGCs or embryos suppresses migration during embryogenesis.

## 4 Discussion

Chicken PGCs exhibit unique characteristics that distinguish them from somatic cells and gametes. During embryogenesis, chicken PGCs migrate through the vascular system to the germinal ridge to colonize the developing gonads and form gametes (Ichikawa and Horiuchi, 2023). Chicken PGCs are rich in glycogen and express high levels of pluripotency and germ cell-specific genes (Altgilbers et al., 2021). Moreover, the morphology, pluripotency, and proliferation can be maintained in chicken PGCs in long-term culture *in vitro* or through cryopreservation (Niu et al., 2024; Ibrahim et al., 2024). Here, we demonstrated that PGCs isolated from embryonic gonads of WLH chicken show typical characteristics, as detected by microscope observation, PAS staining, and immunofluorescence. Furthermore, we visually verified the migration of these PGCs *in vivo* through the blood to gonads. These findings confirmed the successful establishment of WLH gonad-derived PGCs, providing a visual system for further investigating PGC migration and development.

With the advancement of PGC application technology, the migration of injected PGCs from the embryonic bloodstream to the gonadal ridge becomes a critical step in the generation of gene-edited chickens. It is only through the successful colonization of donor PGCs in the embryonic gonads that the edited genes can be transmitted to the next-generation via sexual reproduction. Up to now, the literature on PGC migration remains relatively scarce. Analyses of PGC transcriptome profiles have revealed that key genes in cell adhesion, proliferation, DNA methylation, and histone modification are involved in chicken PGC migration (Huang et al., 2022; Rengaraj et al., 2022). Chemokine stromal cell-derived factor *SDF-1* was found to guide PGCs in chicken embryos (Stebler et al., 2004). A deficiency in the transcription factor *Oct4* suppresses chicken PGC migration and gondadal colonization (Meng et al., 2022). In both sexes and various avian species, the migration and differentiation regulatory networks in chicken PGCs are conserved (Park and Han, 2013).

In the context of gene editing, the efficiency of expressing edited genes in the germline is heavily dependent on the biological characteristics of the transferred PGCs and their accurate migration. If chicken PGCs fail to differentiate or colonize properly, the edited genes may not be incorporated into the developing gametes, rendering the gene editing process ineffective for germline transmission. Therefore, understanding the conditions that affect the biological characteristics and migration of PGCs in the chicken embryo is crucial for the development of gene editing techniques. While the development of chicken embryos is influenced by many environmental factors (Noiva et al., 2014; Boleli and Queiroz, 2012), chicken PGCs and their migration within the embryos may also be affected, particularly under the conditions of heat stress. Our research addresses this gap by providing the first systematic investigation of the impact of hyperthermia on PGC biological characteristics and its migration *in vivo*.

Heat stress conditions in the natural environment can be highly diverse. To determine the threshold and sensitivity of chicken primordial germ cells (PGCs) to hyperthermia, we designed our study to observe significant biological changes and their effects on PGC migration. The heat stress conditions employed were intended to represent severe heat stress events that could naturally occur, such as during heatwaves or power outages in incubation facilities. We found that hyperthermia in cultured chicken PGCs triggers a heat shock response, reduces viability, inhibits germ cell specificity, and modifies pluripotency. Chicken *NANOG*, *POUV*, and *SOX2* contribute to maintaining cell pluripotency and preserving the integrity of PGCs (Naeemipour et al., 2013). In hyperthermic cultured PGCs, *NANOG* and *POUV* were significantly suppressed, while *SOX2* was partially activated. These observations imply that heat weakens the pluripotency of PGCs, and that *SOX2* exhibits a distinct heat response and may also vary in cellular function. Future research should delve deeper into the role of *SOX2*. Besides, we generated the GFP-positive PGCs to visually track PGC migration in chicken embryos, providing a new tool for studying PGC behavior under heat stress conditions. We found that hyperthermia impairs PGC migration. Donor PGCs that were subjected to heat treatment were transplanted into embryonic blood. However, during subsequent incubation, these PGCs failed to colonize the recipient gonads. PGCs cultured *in vitro* are critical for PGC-mediated germline transmission techniques. A number of PGC lines have been successfully established from the blood of early-stage embryos (Idoko-Akoh et al., 2023; Chaipipat et al., 2023; Zhang et al., 2023). Our findings further suggest that the efficiency of producing genetically edited chickens will be significantly reduced when PGCs exposed to high temperatures are used. Moreover, our results provide a reference for maintaining appropriate temperatures when culturing PGCs *in vitro*, and for avoiding the use of PGCs that have been exposed to high temperatures in the preparation of gene-edited chickens.

Besides, we found that donor PGCs in hyperthermic embryos rarely colonized the gonads. Gametes of adult animals stem from gonadal PGCs and determine reproductive potential. The disruption of PGC migration induces sterilization in zebrafish (Wong and Collodi, 2013), the disruption of PGC in embryonic gonads induces sterilization in chicken (Ballantyne et al., 2021; Chen et al., 2023). Therefore, our findings indicate that hyperthermia during early embryogenesis hinders the generation of gene-edited chickens by suppressing PGC gonadal mosaicism. There is a critical need to meticulously control incubation conditions to mitigate heat stress, particularly for recipient eggs, ensuring the genetic integrity of hatchlings and, consequently, the successful acquisition of donor PGC-mediated gene-modified chickens.

In hyperthermic embryos, it remains unclear whether the PGCs themselves or the connection between embryonic gonads and PGCs are affected by hyperthermia, thereby inhibiting PGC migration. The changes in PGC properties and metabolic processes caused by hyperthermia and related to the suppression of PGC migration need further research. It is possible that similar to germ cells, various mechanisms in PGCs, such as apoptosis, DNA damage, and autophagy, are triggered by heat, thereby damaging PGCs and inhibiting migration (Zhang et al., 2012; Durairajanayagam et al., 2015). Moreover, evidence suggests that *HSP90* inhibition in mouse or chicken embryos suppresses PGC migration to gonads (Lejong et al., 2020; Vanmuylder et al., 2009); however, *HSP90* in this study was upregulated. Additionally, it is worth noting that donor PGCs exposed to hyperthermia *in vitro* for 12 h were found to partially surround the ED9 embryonic gonads. This suggests that there may be a threshold duration below which hyperthermia does not adversely impact donor PGC migration. Furthermore, hatching under slightly elevated temperatures enhances chicken heat resistance (Loyau et al., 2015; Yalcin et al., 2022). It is possible that an optimal heat treatment of recipient embryos could both not suppress the generation of donor PGC-mediated gene-edited chickens and enhance their heat resistance.

In conclusion, our study established a PGC line derived from White Leghorn chickens and tracked their *in vivo* migration using fluorescence. Our key discovery was that hyperthermia negatively impacts PGCs and their migration. These findings are pivotal for chicken genome editing, as they can guide the optimization of editing protocols by understanding the thermal thresholds we identified. By doing so, the chimerism rate of gene-edited PGCs within gonads may be elevated, which could subsequently boost the success rate of producing genetically edited chickens. Our work not only aids in the advancement of superior poultry breeds but also holds significant potential for shaping agricultural biotechnology and enhancing food security.

## Data Availability

The raw data supporting the conclusions of this article will be made available by the authors, without undue reservation.
